# The future of biologically inspired next‐generation factories for chemicals

**DOI:** 10.1111/1751-7915.12796

**Published:** 2017-08-14

**Authors:** Claudia Schmidt‐Dannert

**Affiliations:** ^1^ Department of Biochemistry, Molecular Biology and Biophysics University of Minnesota 1479 Gortner Avenue St. Paul MN USA

## Abstract

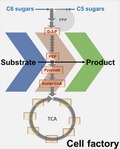

This century is expected to see a drastic acceleration of the field of industrial biology. Biotechnological processes are envisioned to facilitate the production of either high‐value compounds or materials that cannot be made by current methods, or high‐volume chemicals where biological processes are more economical and resource efficient with a reduced environmental impact. Industrial manufacturing of a range of chemicals and materials will utilize both biological and chemical syntheses synergistically. Engineering biology to advance biology has consequently been identified as a top technology priority area in Europe and the United States (Friedman and Ellington, 2015). Yet, shifting manufacturing of chemicals and materials from purely petroleum‐derived chemical synthesis to greener and environmentally friendly biomanufacturing processes that can operate at the same scale and with comparable cost margins is challenging. A major bottleneck is the development of innovative technologies for robust, cost‐efficient and high‐yielding execution of a series of enzyme catalysed conversion reactions that convert one or more molecules into a desired chemical product. This contribution primarily assesses and discusses current and future contributions of microbial technologies as they relate to biomanufacturing towards “ensuring sustainable consumption and production patterns” (goal 12).

Biomanufacturing processes may be performed using cell factories, cell‐free processes or biocatalytic routes (Scheme 1). The cell factory route uses microbial cells and is presently the most advanced technology and preferred route for the large‐scale production of chemicals that require several enzymatic transformation reactions. Microorganisms have a long tradition as cell factories and are used for the large‐scale production of commodity chemicals such as organic acids, amino acids and bioactive compounds like antibiotics. Microbial cells have also been metabolically engineered for the production of many other molecules with applications as fine chemicals, chemical building blocks, fuels, food and feed additives and pharmaceuticals. So far, however, only a limited number of these microbial cell factories have achieved yields and titres at rates that make them economically viable. It turns out, rerouting cellular metabolic networks for the efficient production of, for the cell, undesirable and costly molecules is difficult and requires major engineering efforts that can take a decade or longer (Nielsen and Keasling, 2016; Clomburg *et al*., 2017). Multidisciplinary efforts in science and engineering are required for the realization of a robust and viable bioeconomy.

**Scheme 1:**
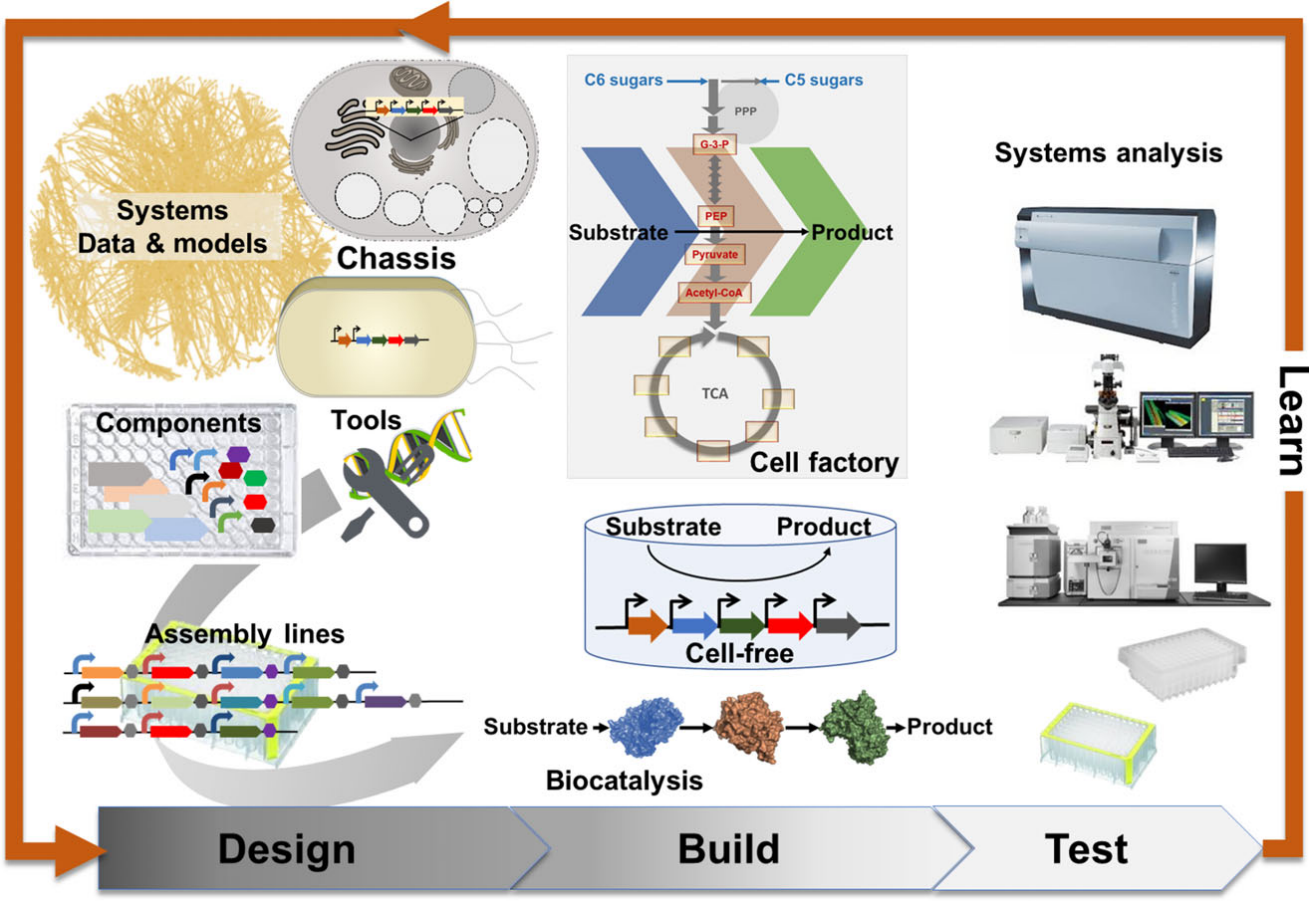
Biofoundries – Integrated biomanufacturing process development platform.

Over the past two decades, we have seen tremendous technical developments in biotechnology that are the foundation for a new area of chemical manufacturing. DNA sequencing has become so inexpensive and readily accessible that now more genome sequences are deposited than individual gene. Similarly, inexpensive DNA synthesis together with efficient, commercially available DNA assembly methods has essentially eliminated classic cloning and instead enabled recoding of sequences and rapid construction and assembly of genetic parts to create gene libraries and pathways. Genome engineering using CRISRP‐Cas9 has revolutionized cellular engineering. In principle, we should therefore be poised to fully exploit the chemical mastery of biology for the engineering of nature inspired chemical factories. In fact, new technologies and strategies are being created and adapted to speed up the microbial strain development process to make chemicals with yields, titres and rates that are industrially relevant.

Biofoundries are developed in academic institutions and industry (Chao *et al*., 2017) that aim to perform iterative design, build, test and learn cycles for strain design in weeks instead of months or years (Scheme 1). Key elements of these platforms are computationally driven systems modelling, *in silico* pathway prediction, creation of genetic part libraries, high‐throughput design and assembly of genetic constructs and analysis of strain libraries. The entire platform requires automation where data from each cycle inform the design of the next cycle until desired strain performance metrics are reached. Interfacing different equipment and analytical instrumentation, however, is a major challenge as is the computational integration and analysis of analytical data (the learning step) to automatically feed the next design, build, test cycle(s).

Current strain design efforts are limited to few well‐characterized chassis organisms and do not take advantage of the large diversity of microorganisms and their unique metabolic capabilities. The establishment of new chassis organisms, however, is tedious and includes, for example, the creation of new genetic and genome editing tools, parts libraries, genome‐scale metabolic models as well as bioprocess conditions. Yet, the development of new chassis organisms will be particularly important for the discovery of new enzyme functions, the conversion of toxic or recalcitrant substrates such as lignocellulose and processes that involve toxic products.

Disruptive engineering approaches are needed that exploit the subcellular organization of microbial cells to access, compartmentalize and reroute precursor pools in order to overcome the resilience of cellular metabolism for higher product titres and yields. Organelles and cellular structures may be repurposed in eukaryotic cells and artificial metabolosomes engineered in bacterial and eukaryotic chassis organisms. Compared with other cellular functions, relatively little is known about the mechanisms that govern the organization of cellular metabolism, including compartmentalization, transport and the dynamics and restructuring of membranes and organelles in microbial cells. Fundamental studies in this area have traditionally been performed by cell biologists but have received little attention from microbial engineers. Only very recently have scientists begun to characterize and engineers systems for the spatial organization of enzymes for *in vitro* and *in vivo* biocatalysis (Quin *et al*., 2017).

Strategies for the rapid design of novel and non‐natural pathways to desired chemical compounds not naturally made by cells will be crucial for shifting chemical manufacturing from petroleum‐based synthesis to biomanufacturing. Bioinformatic strategies are needed for rapid mining of genomic information and especially, genomic dark matter made up of hypothetical and predicted genes, for candidate enzymes with new functions to access new chemistries. Automated extraction of putative enzyme functions works reasonable well for natural products pathways where enzymes are clustered, but not for many other enzymes that are not clustered and with no close homologues. Chemoinformatic approaches together with metabolic modelling are required to design hypothetical pathways that are thermodynamically favourable, carbon efficient and cofactor balanced to serve as guides for enzyme discovery and characterization (Stine *et al*., 2016). Cell‐free approaches are being developed for rapid characterization of enzymes and proto‐typing of pathways (Karim and Jewett, 2016). These strategies will accelerate the design, build, test cycles by taking out the *in vivo* pathway assembly step until suitable candidate enzymes and proto‐type pathways have been identified.

In conclusion, although major strides have been made towards the development of next‐generation cell factories for chemicals biomanufacturing, major hurdles remain to be overcome before these technologies will be cost‐competitive to traditional chemical synthesis. Innovations in computational approaches and cellular engineering, development of strategies for data integration and automation and of new microbial systems are essential for this to happen in the near future. We have created more omics data than can possibly be organized, analysed and used by current human resources. Advanced data visualization platforms with intuitive interaction interfaces will therefore become essential for automated and user‐guided organization and analysis of complex and large multi‐omics data sets for biological engineering purposes. Consequently, raining of the next generation of microbiologists and bioscientists tasked with developing and implementing these biofactories will need to be realigned. Our scientific workforce will require a broad training in synthetic biology, chemistry and bioinformatics and in addition, need to have coding skills and be familiar with, e.g., Python, MathLab and other common software platforms. Considering that developments in biotechnology have outpaced Moore's law in the computer industry and if the field of biotechnology continues to rapidly evolve and innovate, we may see a transition from chemical plants to bio‐based factories within the next 10–15 years. As such, bio‐based factories will contribute and impact several “Sustainable Development Goals”; in addition to goal 12 (sustainable consumption and production patterns), they will “ensure access to affordable, reliable, sustainable and modern energy for all (goal 7)” and “promote sustained, inclusive and sustainable economic growth, full and productive employment and decent work for all (goal 8)”.

## Conflict of Interest

None declared.
